# IGF/IGF-1R signal pathway in pain: a promising therapeutic target

**DOI:** 10.7150/ijbs.84353

**Published:** 2023-07-03

**Authors:** Lulin Ma, Wenjing Zhao, Shiqian Huang, Feng Xu, Yafeng Wang, Daling Deng, Tianhao Zhang, Shaofang Shu, Xiangdong Chen

**Affiliations:** 1 Department of Anesthesiology, Union Hospital, Tongji Medical College, Huazhong University of Science and Technology, Wuhan, 430022, China.; 2 Institute of Anesthesia and Critical Care Medicine, Union Hospital, Tongji Medical College, Huazhong University of Science and Technology, Wuhan, 430022, China.; 3 Key Laboratory of Anesthesiology and Resuscitation (Huazhong University of Science and Technology), Ministry of Education, China.

**Keywords:** Pain, IGF/IGF-1R pathway, Neuronal excitability, Neuroinflammation, Glial cell

## Abstract

Pain, one of the most important problems in the field of medicine and public health, has great research significance. Opioids are still the main drugs to relieve pain now. However, its application is limited due to its obvious side effects. Therefore, it is urgent to develop new drugs to relieve pain. Multiple studies have found that IGF/IGF-1R pathway plays an important role in the occurrence and development of pain. The regulation of IGF/IGF-1R pathway has obvious effect on pain. This review summarized and discussed the therapeutic potential of IGF/IGF-1R signal pathway for pain. It also summarized that IGF/IGF-1R regulates pain by acting on neuronal excitability, neuroinflammation, glial cells, apoptosis, etc. However, its mechanisms of occurrence and development in pain still need further study in the future. In conclusion, although more deep researches are needed, these studies indicate that IGF/IGF-1R signal pathway is a promising therapeutic target for pain.

## 1. Introduction

Pain is a complex biopsychosocial phenomenon, which is redefined as “an unpleasant sensory and emotional experience associated with, or resembling that associated with, actual or potential tissue damage” by the International Association for the Study of Pain (IASP) in 2020^1^. Pain is mainly divided into acute pain and chronic pain according to the time definition. Acute pain, mainly caused by surgery, influences early mobilization as a prerequisite for improving recovery and reducing the risk of complications^2^. Acute severe pain increases the risk of transition to chronic postoperative pain^3^, ^4^ as well as the occurrence of postoperative delirium^5^. Therefore, it is particularly important to control acute pain in time. While chronic pain, as one of the most common reasons adults seek medical care, has a prevalence rate between 11% and 40%, which seriously affects the quality of patients' life^6^. It is estimated that 20.4% (50 million) of American adults suffer from chronic pain^6^. In addition to suffering from pain, chronic pain is also prone to comorbidity such as anxiety, depression and sleep disorders^7^-^10^. The economic cost of chronic pain is enormous. A report released by the Medical Research Institute in 2010 estimated that chronic pain afflicts about one third of Americans, causing medical expenses and productivity losses of 560 to 635 billion dollars each year^11^. Therefore, pain, as one of the most important problems in the field of medicine and public health, has great research significance. To date, the mechanisms of pain have not been clearly studied, so it is urgent to understand the mechanisms of pain in order to conduct better treatment.

Insulin-like growth factor (IGF), the primary effector of the growth hormone (GH) axis, is an anabolic hormone produced mainly in the liver. It can also be produced by local autocrine and paracrine, such as skeletal myogenic cells^12^, ventricular fibroblasts^13^, glial cells^14^. It involves in regulating biological activities related to GH, such as insulin metabolism and cell proliferation, differentiation and apoptosis^15^. Existing studies have found that IGF plays an important role in various human diseases. Several studies show that IGF-1 is closely related to the metabolism of cancer^16^-^19^. The abnormal levels of IGF are associated with increased risk of diabetes^20^-^23^ and cardiovascular disease^24^-^26^. Besides, clinical evidence and animal studies indicate the role of IGF/GH pathway involved in the process of longevity and aging^27^-^29^. More research found that IGF has nutritional and anti-apoptotic effects on neurons and muscle cells^30^-^33^. In addition, whether acute or chronic pain, IGF plays an important role in its occurrence and development^34^, ^35^.

In this review, we emphasized the latest progress in understanding the mechanisms of IGF in acute and chronic pain, focusing on how IGF affects pain through changes in neuronal excitability, neuroinflammation, and glial cells. It clarifies the different roles of IGF in different pain types and different regions, and provides new ideas and targets for future pain treatment.

## 2. The overview of IGF/IGF-1R pathway

IGF system includes an ancient peptide family, which involves mammalian growth, development and metabolism, as well as cell processes, such as proliferation, survival, cell migration and differentiation. IGF family includes three known ligands (IGF-I, IGF-2 and insulin), six characteristic insulin-like growth factor binding proteins (IGFBP-1-6) and cell surface receptors (IGF-1 receptor, insulin receptor and IGF-II mannose 6-phosphate receptor) that mediate ligand action^36^-^38^. IGF acts through circulation and local secretion^39^. These molecules constitute a complex autocrine/paracrine network to regulate cell proliferation, survival and differentiation^39^. Among them, IGF-1 and IGF-2 are reported to be related to pain at present. IGF-1 is a single polypeptide chain consisting of 70 amino acids, which has a binding affinity to IGF-IR 100-fold higher than insulin. IGF-2 is composed of 180 amino acids. It also binds to IGF-IR with a binding affinity comparable to IGF-1^40^, ^41^.

IGF-1 and IGF-2 are mainly mediated by IGF receptor type 1 (IGF-1R) to regulate pain. IGF-1R was first proved to exist in 1974^42^. IGF-1R can be shown by SDS gel electrophoresis and consists of two α and two β chains linked together by disulfide bonds^40^, ^43^. When expressed in the presence of monensin, an inhibitor of posttranslational processing, the IGF-1R was proved to be synthesized as a 180-kDa precursor, which is glycosylated, dimerized and proteolytically to produce the mature α2β2 receptors. The next key finding is that IGF-1R, like insulin receptor (IR), is a tyrosine kinase which is activated and autophosphorylated following IGF binding^44^. The IGF-1R has a similar structure to the insulin receptor. The insulin receptor (and related IGF-1R) is a covalent dimer transmembrane allosteric enzyme^45^. IGF-1R is a member of the transmembrane tyrosine kinase family, including IR and orphan insulin receptor related receptor (IRR). It binds IGF-1 with high affinity and starts the physiological response to the ligand *in vivo*^46^. IGF-1R also binds to IGF-2, plays a part in the mitogenic effect of this polypeptide during fetal development^47^. The extracellular α-subunit forms IGF-1 binding pockets, while the intracellular domain (amino acids 956-1256) of the membrane-spanning β-subunit contains a kinase domain^48^, ^49^. Once activation, the tyrosine kinase activity of IGF-1R results in a continuous phosphorylation event at residues Y1135, Y1131, and Y1136, producing conformational changes in β subunits to create conformational changes occur in subunits, creating docking sites for downstream signal molecules and their phosphorylation^49^. The activation of IGF-1R triggers multiple signal cascades in a cell context dependent manner to regulate multiple cellular functions from proliferation and survival to differentiation into specific cell lineages^39^.

## 3. The relationship between IGF/IGF-1R and pain

### 3.1. The expression of IGF/IGF-1R in pain related regions

Multiple evidence indicates that IGF/IGF-1R can be expressed in dorsal root ganglion (DRG)^50^, ^51^, spinal dorsal horn^52^, ^53^ and brain^54^-^56^. These areas are closely related to pain. Dai et al found that IGF-1 immunoreactive products showed strongly stained brown deposits in the cytoplasm of large, medium and small DRG neurons. In layer II of L3 and L6 spinal cord, IGF-1 immunoreactive products were found in the nuclei of some neurons and glial cells^52^. IGF-1 and its receptor are preferentially expressed in DRG neurons harvested from sciatic nerve axotomy and streptozotocin (STZ) induced painful neuropathy in diabetes^57^. Besides, Takayama also reported that colocalization of IGF-1 in DRG is expressed in the neuronal bodies and fibers. IGF-1 is collocated with neuron, but not with satellite glial cell (SGC) of DRG in a rat disc herniation model^58^. However, single cell RNA (scRNA) sequencing and *in situ* hybridization analysis showed high expression of endogenous IGF-1 in non peptidergic neurons and SGC of DRG^59^, ^60^. IGF-1 expression in cerebral cortex and DRG was higher than that in sciatic nerve in mice^59^, ^60^. Besides, IGF-1R also widely expressed in hippocampus^61^-^63^ and frontal cortex^64^-^66^. In summary, IGF/IGF-1R are widely expressed in sites closely related to pain (DRG, spinal cord and brain), so we reasonably speculate that they are closely related to the occurrence and development of pain.

### 3.2. The role of IGF/IGF-1R in pain

#### 3.2.1. The IGF/IGF-1R pathway involved in aggravating acute pain

IGF/IGF-1R pathway plays an important role in acute pain (Table 1). Local administration of IGF-1 induces thermal and mechanical pain hypersensitivity in a dose-dependent manner, which is attenuated by inhibition of IGF-1R inhibitor picropodophyllin (PPP, 50 μg)^34^. In addition, the author also found that after plantar incision, the level of IGF-1 in tissues, but not in plasma, increased significantly. IGF-1R inhibitor (PPP, 50 μg) successfully alleviated mechanical allodynia, thermal hyperalgesia and spontaneous pain behavior observed after plantar incision^34^. Another study also showed that intraplantar recombinant IGF-1 (rIGF-1, 50 μg/kg) developed hyperalgesia 2 hours later in mice. This IGF-1-induced hypersensitivity response is attenuated by pretreatment with IGF-1R antagonists (JB1, 6 μg)^67^. Clinical experiments also found that the serum IGF-2 level in patients with acute myofascial pain syndrome (MPS) was lower than that in healthy control group, with no gender difference, while the IGF-1 level in each group had no difference^68^. Above all, the dysregulation of IGF/IGF-1R can promote the occurrence of a variety of acute pain, and inhibition of their expression can relieve acute pain.

#### 3.2.2. The IGF/IGF-1R pathway involved in relieving acute pain

However, Takemura et al found that IGF-1 expression increased in paw, but did not increase in DRG after plantar incision. Plantar injection of IGF-1 increased the expression of G-protein-coupled receptor kinase 2 (GRK2) in ipsilateral DRG, which can relive the mechanical hyperalgesia. The application of IGF-1R inhibitor (250 µg/kg and 500 µg/kg) prevented the induction of GRK2 and the regression of hyperalgesia after plantar incision^69^ (Table 2). The reason why this study has different results from the above studies that Miura conducted may be closely related to the different observation time. This experiment mainly focuses on the state 7 days after the incision pain, while the above research focuses on the pain within 3 days after the incision.

#### 3.2.3. The IGF/IGF-1R pathway involved in aggravating chronic pain

There are also research findings reported that the IGF/IGF-1R signaling pathway can cause hyperalgesia in chronic pain (Table 1). IGF-1 increases T-type channel currents through the IGF-1R that is coupled with a G protein-dependent protein kinase C-α (PKC-α) pathway, thereby increasing the excitability and sensitivity of DRG neurons to induce pain^70^. Under complete Freund's adjuvant (CFA) induced chronic inflammatory pain conditions, T-type Cav3.2 channel at least partially mediates the pain promotion through IGF-1/IGF-1R pathway in DRG^71^. Besides, IGF-1 activates IGF-1R to induce neuronal hyperexcitability in small trigeminal ganglion (TG) neurons, decreasing A-type K+ currents (IA) to enhance the sensitivity of pain^72^.

Furthermore, IGF-1 in tibia bone marrow was increased in metastasized bone cancer pain model, while intraperitoneal injection IGF-1R inhibitor (PPP, 20 mg/kg/12 h) *in vivo* can reverse mechanical allodynia and thermal hyperalgesia in rats with bone cancer pain^73^. Then the up-regulation of IGF-1 may be the key factor of painful radiculopathy induced by mechanical factors in a rat lumbar disc herniation (LDH) model^74^ ^58^. IGF-1 knockdown leads to abnormal reduction of mechanical pain^58^. Subcutaneous injection of IGF-1 (2 mg/kg) induced a significant allodynia in treatment-induced painful neuropathy of diabetes (TIND)^75^. In addition, orofacial nerve injury (IONI) induced neuropathic pain can up-regulate the IGF-1, while subcutaneous injection of IGF-1 neutralizing antibody can recover the mechanical head withdrawal reflex thresholds (MHWTs) significantly^76^. Forster et al demonstrated that in a vitro model of endometriosis associated macrophages, disease modified macrophages showed increased expression of IGF-1, and confirmed the expression of damaged resident macrophages in mice. IGF-1R inhibition (linsitinib, 40 mg/kg) attenuates endometriosis induced hyperalgesia in mice^77^. Interestingly, abnormal IGF-1/IGF-1R signaling in spinal cord contributes to chronic constriction injury (CCI) induced neuropathic pain and IGF-1R antagonism (nvp-aew54, 15 μg/day) or IGF-1 neutralizing antibody (1 μg/day) can reduce pain behavior induced by CCI^78^. Similar to CCI, intrathecal administration of IGF-2 siRNA significantly inhibited spared nerve injury (SNI) induced neuropathic pain by inhibiting the expression of IGF-2 in the spinal cord^79^. Moreover, the treatment of pulsed radiofrequency (PRF) also alleviated neuropathic pain caused by SNI through downregulating the expression of IGF-2 in spinal dorsal horn^80^. Therefore, in multiple animal models of chronic pain, IGF/IGF-1R pathway can aggravate chronic pain.

Clinical data found that IGF-1 in peritoneal fluid (PF) significantly increased in patients with endometriosis, and its concentration was positively correlated with pain score^77^. Besides, in patients with breast cancer, increased IGF-1 concentration was reported new onset or worsening pain^81^. Specifically, the IGF-1: IGFBP-3 ratio increased in patients who experienced new pain episodes or pain exacerbations, while it decreased statistically significantly in patients who did not experience new pain or pain exacerbations^81^. Similar to the results of animal experiments, the high expression of IGF-1 in patients strongly associated with increased pain score. So, both animal and clinical experiments suggest that IGF/IGF-1R can aggravate chronic pain.

#### 3.2.4. The IGF/IGF-1R pathway involved in relieving chronic pain

More studies have shown that IGF/IGF-1R signaling pathway is involved in the occurrence and development of chronic pain (Table 2). Kohno et al found that 21 days after peripheral nerve injury (PNI), the up-regulation of IGF-1 in CD11c^+^ microglia of the spinal dorsal horn can alleviate neuropathic pain, while knockout of IGF-1 in CD11c^+^microglia can make neuropathic pain lasts for at least 56 days in mice^35^. Pain thresholds had recovered to baseline on the 35th day after PNI, and intrathecal injection of IGF1-neutralizing antibody could cause pain recurrence^35^. In addition, in the rat neuropathic pain model of chemotherapy induced peripheral neuropathy (CIPN), the expression of IGF-1 protein in the spinal cord was significantly reduced. Intravenous or intraperitoneal injection of rIGF-1 (1 μg) alleviated pain like behavior induced by chemotherapy^82^. In painful diabetic neuropathy (PDN) rat model, subcutaneous injection of IGF-1 (2.5mg/kg) can relieve neuropathic pain^83^. Nasal treatment with IGF-1 can relieve migraine-related pain in animal models^84^. Chu et al reported that the STZ treated mice developed hyperalgesia at the early stage, followed by hypoalgesia, and the systemic IGF-1 level was significantly reduced. Increasing circulating IGF-1 can significantly relieve neuropathic pain in diabetes^85^. Inhibition of IGF-1 (intrathecal injection of LV shIGF-1 lentivirus vector) can aggravate cord compression injury (SCI) induced neuropathic pain^86^. In addition, painful diabetic neuropathy significantly reduces the expression of IGF-1 in spinal cord. Intraperitoneal injection rIGF-1(1 μg/d) in mice alleviates pain-like behavior induced by diabetes^87^. Interestingly, electroacupuncture treatment can relieve neuropathic pain following nerve injury by activating the IGF-1 pathway^88^. Bagriyanik et al found that resveratrol (RVT) reduced CCI induced damage through IGF-1 immunoreactivity^89^.

Clinical experiments also show that serum IGF-1 level is related to pain. Low serum IGF-1 levels are associated with pain in patients with fibromyalgia syndrome (FMS)^90^-^92^. Koca et al reported that FMS patients showed lower IGF-1 serum levels, but with no related to disease severity^93^. However, another research found that women with FMS and low IGF-1 levels improved their overall symptoms and number of pain points after receiving daily growth hormone therapy for 9 months, indicating that increased IGF-1 level in serum can relieve FMS^94^. In a lasting 12-month study, the average number of pain points (pairs) decreased by 60% after human growth hormone treatment for FMS patients with low level IGF-1^90^. Besides, a study recruited a total of 493 FMS patients with low IGF-1 to receive the growth hormone treatment. Twelve to eighteen months after stopping the treatment, the growth hormone treatment still had analgesic effect, showing its sustained action over time^95^. All of the above studies suggest that IGF/IGF-1R can relieve chronic pain. However, it has also been found in several reports to aggravate chronic pain. The reasons for the opposite results are mainly due to the different animal models, different observation time windows and different observation sites in each study. Therefore, more studies are needed to explore and find the pattern for the symptomatic management of pain.

## 4. Mechanisms of IGF-1/IGF-1R pathway related to pain

### 4.1. Relationship between IGF -1/IGF-1R pathway and neuronal excitability

DRG neurons that diameter less than 30 μM are mainly involved in nociceptive signal transduction^96^, ^97^. The incubation of IGF-1 (100 nM) enhanced the peak amplitude of T-type calcium channel current in small DRG neurons, while the amplitude was only partly recovered in 3 minutes after IGF-1 was washed away^70^. However, the current was eliminated when JB-1 (a selective IGF-1R antagonist, 1 μM) was used, indicating that IGF-1R is involved in IGF-1 mediating the influence on T- type calcium channel current in small DRG neurons. The author also found that JB-1 mainly affects the current of Cav3.2 in DRG neurons^70^. T-type Cav3.2 calcium channel belongs to low voltage activated Ca^2+^ channel and is an important contributor to nociceptive signal transmission in primary afferent pain pathway^98^, ^99^. Similar to this study, in another CFA induced chronic inflammatory pain mice model, T-type Cav3.2 channel can facilitate and amplify pain signals through the activation of IGF-1/ IGF-1R^71^. Lin et al also found that the expression of Cav3.2 and IGF-1R in lumbar DRG was up-regulated after sciatic neurotomy, indicating the interaction of T-type Cav3.2 channel and IGF-1 can contribute to pain hypersensitivity in primary sensory nerves^100^. Besides, there are two main types of outward voltage gated K^+^ channel (Kv) currents in nociceptive neurons to induced pain: I_A_ and I_DR_^101^-^103^. Bath application of IGF-1 (0.1 μM) inhibited I_A_ in small TG neurons. After the elution of IGF-1, the amplitude of I_A_ partially recovered to the pretreatment level within 5 minutes. However, treating TG neurons with selective IGF-1R antagonist PQ-401 (10 μM) completely eliminated IGF-1 (0.1 μM) induced I_A_ reduction^72^. Transient receptor potential vanilloid 1 (TRPV1) current density increased in acutely isolated DRG neurons with MRMT-1 bone cancer pain in rats. Then the authors incubated DRG neurons with IGF-1 (30 or 100 ng/mL), which could increase the expression of total and membrane TRPV1 protein, indicating that IGF-1 can aggravate pain by increasing TRPV1 current^73^.

However, subcutaneous injections of IGF-1 (2.5 mg/kg) can relieve the pain behaviors induced by PDN, and reverse the hyperactivity of neurons in the spinal cord and ventrolateral PAG^83^. Therefore, IGF-1/IGF-1R pathway can regulate pain by regulating the excitability of nociceptors and spinal cord. The opposite effect of IGF-1/IGF-1R pathway in pain may be related to different pain models or different effect regions. The peripheral IGF-1/IGF-1R pathway may play a role in promoting pain, while the central (spinal cord) IGF-1/IGF-1R pathway may play a role in inhibiting pain.

### 4.2. Relationship between IGF /IGF-1R pathway and neuroinflammation as well as glial cells

#### 4.2.1 IGF /IGF-1R pathway and neuroinflammation

P38, extracellular signal-regulated kinase (Erk), and Jun N-terminal kinase (JNK) are the family of mitogen-activated protein kinase (MAPK)^104^, ^105^. These molecules transfer from the cytoplasm to the nucleus and activate many transcription factors, such as NF-kappaB (NF- κB) and activator protein-1 (AP-1), which can promote the release of various molecules, including p16, p21, IL-6 and interlukin-18 (IL-8). They can thus cause inflammation in pain related disease^106^, ^107^. Intraplantar injection of rIGF-1 (50 μg/kg) increase the expression of c-raf and p-ERK, while JB1 (6 μg/mouse) pretreatment significantly reduced p-ERK and c-raf levels in DRG^67^. Besides, PRF can inhibit ERK1/2 activity in microglia by down-regulating IGF-2 to relieve the neuropathic pain^80^. Antagonism of spinal IGF-1R (nvp-aew541, 15 μg) suppressed the overexpression of IL-1β, TNF-α, and IL-6 that induced by up-regulation of mTOR in CCI neuropathic pain^78^.

Intrathecal or intraperitoneal injection of rIGF-1 (1 μg) inhibits neuropathic pain induced by oxaliplatin therapy, which can reduce the expression of IL-17A and TNF- α in spinal cord^82^. In addition, downregulation of miR-130a-3p increased the expression of IGF-1 and IGF-1R in spinal cord. When the expression of IGF-1 and IGF-1R were up-regulated, they can suppress the expression of NF- κB phosphorylation and IL-1β, IL-6 and TNF-α in SCI rats^86^.

#### 4.2.2 IGF /IGF-1R pathway and glial cell

It is well known that the neuroinflammatory response is almostly caused by exactly glial cells. Therefore, activation of glial cells is also closely associated with neuroinflammation. A study reported that IONI promoted macrophage infiltration into damaged ION and the corresponding TG. On the third day after IONI, the amount of IGF-1 released by macrophages in ION and TG increased significantly. In addition, subcutaneous injection of neutralization of IGF-1 (20 ng) partially inhibited the mechanical hypersensitivity induced by IONI^76^. In CCI mice, the number of astrocytes collocated with IGF-1 in the ipsilateral spinal dorsal horn was significantly more than that of the contralateral side. Intrathecal injection of IGF-1 neutralizing antibody (1 μg/day, 3 days) inhibits activation of spinal astrocytes. Besides, inhibition of IGF-1 expression also suppressed the expression of spinal cord neuroinflammation such as TNF-a, IL-B and IL-6^78^. In endometriosis induced pain mice model, the mRNA expressions of Cox-2 and TNF-α that macrophages released in spinal cord increased and these levels decreased after depletion of macrophages with liposomal clodronate^77^.

The Science article found that PNI mice with exhausted spinal cord CD11c^+^microglia failed to recover spontaneously from this hypersensitivity reaction. Down-regulation of IGF-1 signal once again shows the pain hypersensitivity from the recovery. In mice with pain recovery, the depletion of CD11c^+^microglia or the interruption of IGF-1 signal led to the recurrence of pain hypersensitivity^35^. In PDN mice model, IGF-1 expression in spinal microglia decreased significantly. Intrathecal injection of rIGF-1 (1 μg/d, 3 days) prevents M1 microglia polarization (iNOS+Iba-1+microglia) and reduces the corresponding M1 neuroinflammation, such as iNOS, IL-1β and TNF-α^87^. In summary, IGF/IGF-1R can widely affect the occurrence and development of pain by regulating glial cells and neuroinflammation. Therefore, the intervention of IGF/IGF-1R pathway may regulate pain by regulating neuroinflammation.

### 4.3. Relationship between IGF /IGF-1R pathway and other aspects

Bath application of IGF-1 (0.1 μM) reduced I_A_ in small TG neurons. When selective IGF-1R antagonist PQ-401 (10 μM) was used to treat the TG neurons, it could completely eliminate IGF-1 induced I_A_ reduction. While when TG neurons were pretreated with phosphatidylinositol-3-kinase (PI3K) inhibitor wortmannin (1 μM), IGF-1 had no impact on I_A_. Another PI3K inhibitor LY294002 (20 μM) had the similar results, indicating that PI3K contributes to IGF-1R mediated IA reduction^72^. PI3K, known as a conservative signal transduction enzyme family, is involved in regulating cell growth, cycle entry, migration and survival^108^, ^109^. Protein kinase B (PKB/AKT), was activated via PI3K pathway^110^, also plays an important role in the pain regulation of IGF/ IGF-1R pathway. Miura et al reported that after plantar incision, the expression of phosphorylated Akt in DRG neurons increased significantly and was inhibited by the single administration of picropodophyllin (an inhibition of IGF-1R, 50 μg) to the plantar skins. The increase of IGF-1 production in skin tissues sensitizes primary afferent neurons through IGF-1R/Akt pathway to promote pain hypersensitivity after tissue injury^34^.

Besides, dynamic regulation of GRK2 negatively regulates nociception in primary afferent neurons. Plantar injection of IGF-1 (1 μg) increased the expression of GRK2 in ipsilateral DRG. The application of IGF-1R inhibitor (picropodophyllin, 250 µg/kg) prevented the induction of GRK2 and the regression of hyperalgesia after plantar incision, indicating that the induction of GRK2 expression driven by tissue IGF-1 has an effective analgesic effect^69^. Hu et al reported that the expression levels of IGF-1, pPI3K and pAkt down-regulated in DRG, and overexpression of IGF-1 reduced the pain intensity by activating PI3K/Akt pathway in rat model of adjacent dorsal root ganglionectomies^88^. Furthermore, after PNI, AXL mRNA was expressed in CD11c^high^ SDH microglia, which is a member of the TAM (TYRO3, AXL, and MERTK) family of tyrosine kinase receptors involved in the engulfment of myelin debris. AXL participates in the appearance of CD11 c^high^ spinal dorsal horn microglia to induce the expression of IGF-1, promoting the relief of PNI induced neuropathic pain^35^. Therefore, IGF/IGF-1R regulates pain through a variety of ways. In addition to participating in the regulation of neuronal excitability and neuroinflammation, it also regulates pain in other ways, such as apoptosis, autophagy, etc.

## 5. Discussion and perspective

**Figure 1:**
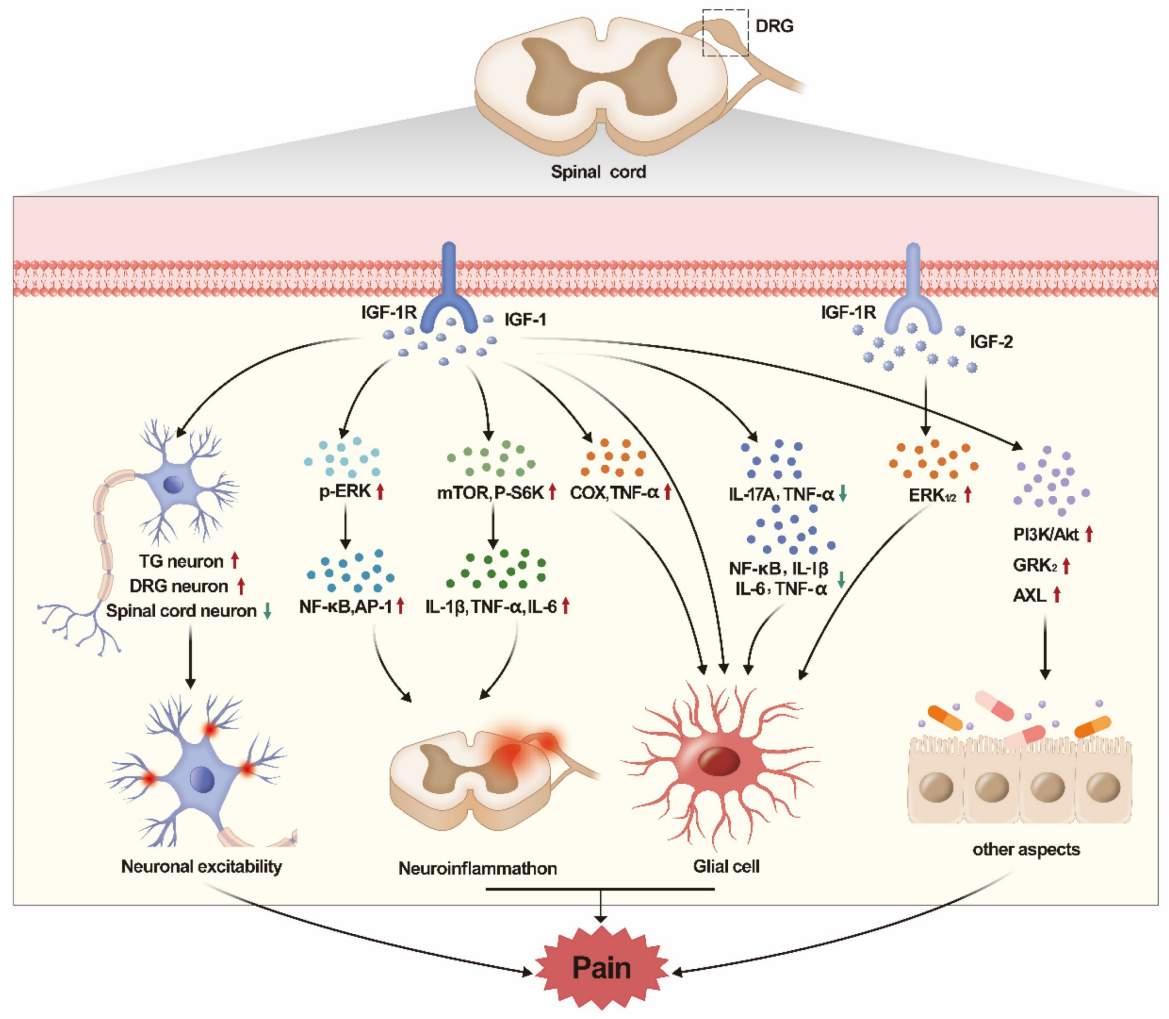
The graphic image of this review. The regulation of IGF/IGF-1R pathway in DRG and spinal cord has obvious effect on pain. The IGF/IGF-1R regulates pain by acting on neuronal excitability, neuroinflammation, glial cells, apoptosis, etc.

In this review, we summarized the role of IGF/IGF-1R pathway in various types of pain. It was found that IGF/IGF-1R played different roles in different types of pain. Besides, IGF/IGF-1R also played different roles in different regions. The peripheral IGF-1/IGF-1R pathway mainly plays a role in promoting pain, while the central (spinal cord) IGF-1/IGF-1R pathway mainly play a role in relieving pain. The mechanisms of IGF are different in different pain models. IGF-1/IGF-1R pathway increases excitability and pain sensitivity in peripheral neurons, but it can reverse the excitability of neurons at the spinal cord, thereby reducing pain sensitivity. Besides, another main mechanism of IGF-1/IGF-1R pathway is to affect pain by regulating glial cells and neuroinflammation. However, IGF-1/IGF-1R has different effects on inflammation in different pain models. Of course, the IGF-1/IGF-1R pathway can also regulate pain through autophagy, demyelination and PI3K/AKT pathway. At last, few studies reported the relationship between IGF-2 and pain (see Fig. 1 for summary).

Currently, the main researches mainly focused on the relationship between IGF-1 and pain. Researches on IGF and pain mainly focus on peripheral (DRG, TG) and lower central (spinal cord) system. However, IGF-1R is expressed not only in DRG^51^ and spinal cord^111^, but also in brain such as hippocampus and cortex^111^-^117^. Chen et al indicated that rufinamide can improve cognitive function and increase neurogenesis in the hippocampus of the aged gerbil by increasing the IGF-1, IGF-1R and p-CREB expressions^63^. IGF-1R signals were damaged in Alzheimer's disease (AD) neurons in temporal cortex, which indicated that degenerated neurons in AD might be resistant to IGF-1R/IR signals^118^. In hippocampus, IGF-1 signaling axis can regulate traumatic brain injury (TBI) induced damage (cognitive as well as cellular)^119^. Therefore, IGF-1/IGF-1R also plays a regulatory role in various diseases in the brain. Pain, especially chronic pain, is closely related to the brain^120^-^122^. We speculate that the IGF-1/IGF-1R signal axis in the brain is also involved in pain regulation, which needs further research to confirm. Combined with the role of IGF/IGF-1R in DRG and spinal cord, IGF/IGF-1R in different regions of the brain may play different roles in regulating pain, which also needs further research in the future.

Besides, combined with existing research findings, it was found that the regulation of IGF/IGF-1R pathway on pain is closely related to the time of pain occurrence. In the acute phase of pain, IGF/IGF-1R pathway mainly plays a role in promoting pain. While with the extension of time, IGF/IGF-1R pathway may alleviate pain. It suggests that IGF/IGF-1R may play different roles in different stages of pain occurrence and development. This is also the research that needs to be continued in the future.

Last, although some studies have confirmed that IGF-2 also played a regulatory role in pain, there are few studies at present about the relationship between them. Uchimura et al reported that IGF-2 inhibited the expression of IL-1β induced cartilage matrix loss and promoted cartilage integrity in experimental osteoarthritis (OA)^123^. In OA cartilage, the IGF-1, IGF-2, IGF-1R and IRS1 in the degenerated area declined compared with the reserved area^124^. Multiple study also indicated the role of IGF-2 in OA^125^-^127^. Therefore, IGF-2 may also play an important role in pain, which is also the direction for the further researches.

## 6. Conclusion

Multiple studies have found that IGF/IGF-1R pathway plays an important role in the occurrence and development of pain. IGF/IGF-1R regulates pain by acting on neuronal excitability, neuroinflammation, glial cells, apoptosis, etc. In conclusion, although more deep researches are needed in the future, these studies indicate that IGF/IGF-1R signal pathway is a promising therapeutic target for pain.

## Figures and Tables

**Table 1:** The pain model, expression, localization of the IGF/IGF-1R aggravating the pain.

IGF/IGF-1R	Pain model	Expression	Localization	Reference
IGF/IGF-1R	DRG neuron/CFA	DRG ↑	Neuron	70
IGF-1R	CFA	DRG -	Neuron	71
IGF-1/IGF-1R	bone cancer pain.	tibia bone cavity ↑	Neuron	73
IGF-1	lumbar disc herniation (LDH) model	DRG ↑	Neuron	58 74
IGF-1	infraorbital nerve injury (IONI) induced neuropathic pain	TG ↑ infraorbital nerve ↑	Macrophage Satellite glial cell	76
IGF-1/IGF-1R	Spinal cord injury induced neuropathic pain	-	-	-
IGF-1/IGF-1R	pain associated with endometriosis	peritoneal fluid (PF) ↑	Macrophage (IBA1)	77
IGF-1/IGF-1R	TG neuron	TG	Neuron	73
IGF-1/IGF-1R	chronic constriction injury (CCI)	Spinal cord ↑	Microglia (IGF-1) Astrocyte (IGF-1) Neuron (IGF-1) Neuron (IGF-1R)	78
IGF-1/ IGF-1R	plantar incision induced pain	DRG ↑	Neuron (IGF-1R) Satellite glial cell (IGF-1R)	34
IGF-1/IGF-1R	Intraplantar recombinant (r) IGF-1	DRG (IGF-1R) ↑	TRPV1 neuron	67
IGF-2	spared nerve injury (SNI) induced neuropathic pain	Spinal cord ↑	Neuron CD11b microglia	79 80

**Table 2:** The pain model, expression, localization of the IGF/IGF-1R relieving the pain.

IGF/IGF-1R	Pain model	Expression	Localization	Reference
IGF-1/ IGF-1R	plantar incision induced pain	Plantar tissue ↑	DRG	69
IGF-1	spinal nerve injury model	Spinal cord ↑	CD11c^+^ microglia	35
IGF-1/IGF-1R	Chemotherapy-induced peripheral neuropathy (CIPN)	spinal cord↓ spinal cord neuron activity -	IGF-1 (astrocyte) IGF-1R (Neuron) -	82 83
IGF-1	Diabetic peripheral neuropathy (DPN) induced neuropathic pain	Serum ↓ spinal cord ↓	- astrocyte Neuron microglia (major)	85 87
IGF-1	spinal cord compression injury (SCI) induced neuropathic pain	DRG -	-	86
IGF-1	neuropathic pain following deafferentation injury	DRG -	-	88

## Abbreviations

AD: Alzheimer's disease
AP-1: Activator protein-1
CCI: Chronic constriction injury
CFA: Complete Freund's adjuvant
CIPN: Chemotherapy induced peripheral neuropathy
DRG: Dorsal root ganglion
Erk: Extracellular signal-regulated kinase
FMS: Fibromyalgia syndrome
GH: Growth hormone
GRK2: G-protein-coupled receptor kinase 2
IASP: International Association for the Study of Pain
IGF: Insulin-like growth factor
IGFBP-1-6: Insulin-like growth factor binding proteins-1-6
IGF-1R: Insulin-like growth factor receptor type 1
IL-18: Interlukin-18
IONI: Orofacial nerve injury
IR: Insulin receptor
IRR: Insulin receptor related receptor
JNK: Jun N-terminal kinase
LDH: Lumbar disc herniation
MAPK: Mitogen-activated protein kinase
MHWTs: Mechanical head withdrawal reflex thresholds
MPS: Myofascial pain syndrome
NF- ΚB: NF-kappaB
OA: Osteoarthritis
PDN: Painful diabetic neuropathy
PF: Peritoneal fluid
PI3K: Phosphatidylinositol-3-kinase
PIND: Painful neuropathy of diabetes
PKC-α: Protein kinase C-α
PKB/AKT: Protein kinase B
PNI: Peripheral nerve injury
PPP: Picropodophyllin
PRF: Pulsed radiofrequency
rIGF-1: Recombinant IGF-1
SCI: Spinal cord compression injury
scRNA: Single cell RNA
SGC: Satellite glial cell
SNI: Spared nerve injury
STZ: Streptozotocin
TBI: Traumatic brain injury
TG: Trigeminal ganglion
TIND: Treatment-induced painful neuropathy of diabetes
TRPV1: Transient receptor potential vanilloid 1
RVT: Resveratrol
